# Developing a Buruli ulcer community of practice in Bankim, Cameroon: A model for Buruli ulcer outreach in Africa

**DOI:** 10.1371/journal.pntd.0006238

**Published:** 2018-03-27

**Authors:** Paschal Kum Awah, Alphonse Um Boock, Ferdinand Mou, Joseph Tohnain Koin, Evaristus Mbah Anye, Djeunga Noumen, Mark Nichter, Stop Buruli Consortium

**Affiliations:** 1 Department of Anthropology, Faculty of Art, Letters and Social Sciences, University of Yaoundé I, Yaoundé, Cameroon; 2 Program Unit FAIRMED, Bern, Switzerland; 3 Monitoring & Evaluation Unit FAIRMED, Yaoundé, Cameroon; 4 Bankim Health District, Ministry of Health, Adamaoua Region, Cameroon; 5 School of Anthropology, University of Arizona, Tucson, United States of America; University of Tennessee, UNITED STATES

## Abstract

**Background:**

In the Cameroon, previous efforts to identify Buruli ulcer (BU) through the mobilization of community health workers (CHWs) yielded poor results. In this paper, we describe the successful creation of a BU community of practice (BUCOP) in Bankim, Cameroon composed of hospital staff, former patients, CHWs, and traditional healers.

**Methods and principle findings:**

All seven stages of a well-defined formative research process were conducted during three phases of research carried out by a team of social scientists working closely with Bankim hospital staff. Phase one ethnographic research generated interventions tested in a phase two proof of concept study followed by a three- year pilot project. In phase three the pilot project was evaluated. An outcome evaluation documented a significant rise in BU detection, especially category I cases, and a shift in case referral. Trained CHW and traditional healers initially referred most suspected cases of BU to Bankim hospital. Over time, household members exposed to an innovative and culturally sensitive outreach education program referred the greatest number of suspected cases. Laboratory confirmation of suspected BU cases referred by community stakeholders was above 30%. An impact and process evaluation found that sustained collaboration between health staff, CHWs, and traditional healers had been achieved. CHWs came to play a more active role in organizing BU outreach activities, which increased their social status. Traditional healers found they gained more from collaboration than they lost from referral.

**Conclusion/ Significance:**

Setting up lines of communication, and promoting collaboration and trust between community stakeholders and health staff is essential to the control of neglected tropical diseases. It is also essential to health system strengthening and emerging disease preparedness. The BUCOP model described in this paper holds great promise for bringing communities together to solve pressing health problems in a culturally sensitive manner.

## Author summary

Buruli ulcer (BU) is a neglected tropical disease primarily found in West Africa largely effecting the rural poor. BU has a known cause and cure, but an unknown route of transmission and a poorly understood incubation period. If not treated early and in a timely manner, BU often progresses to an advanced state requiring surgery and prolonged wound care. In the Cameroon, previous efforts to mobilize community health workers and educate community members to identify cases of BU yielded poor results. In this paper, we describe steps undertaken to create a successful BU community of practice (BUCOP) composed of community stakeholders working in concert with clinic staff. The success of the BUCOP was measured in terms of numbers of suspected BU cases referred and confirmed, a decline in treatment drop out, and sustained collaboration among stakeholders both during and following the pilot project. Pilot project success is attributed to an innovative and culturally sensitive approach to BU outreach education, increased levels of patient assistance, and mutual respect among BUCOP members for what each stakeholder contributed to BU detection, treatment, psychosocial support, and spiritual protection.

## Introduction

Buruli ulcer (BU) is one of several neglected tropical skin diseases that afflict the rural population of sub-Saharan Africa, especially the poor living in areas with limited access to health infrastructure [1,2]. BU stands out as one of the most disabling of all neglected tropical diseases. The large majority of cases of BU have been identified in West Africa, particularly the countries of Benin, Cameroon, the Democratic Republic of the Congo, Cote D’Ivoire, Ghana, and Nigeria [3].

BU is caused by *Mycobacterium ulcerans* (MU), a microorganism belonging to the same genus of bacteria as tuberculosis and leprosy. BU has a known cause and cure, but an unknown route(s) of transmission and poorly understood incubation period [4, 5, 6 7]. BU manifests as necrotizing cutaneous lesions. Thirty-five percent of lesions are located on the upper limbs, 55% on the lower limbs, and 10% on other body parts [5]. In Africa, about 48% of those affected with BU are children under 15 years of age [4], and males and females are affected equally.

If not treated early and in a timely manner, BU often, but not always, progresses to an advanced state requiring prolonged wound care and skin grafting. If left untreated or treated late, BU does not kill, but may render the afflicted permanently disabled. Early diagnosis and treatment are the only ways to minimize morbidity and prevent disability [8]. Notably, at present most cases (68%) of BU are diagnosed at a late stage—categories II and III of the disease [9]. Beyond non-identification in its early stages, and delay in seeking care, there is some evidence suggesting that distinct phenotypes of BU may be more likely to progress to severe forms [10].

Prior to 2005, when effective antibiotic treatment was discovered for treating early stages of BU, all cases required surgery. Antibiotic treatment with streptomycin injections and oral rifampicin for 56 days proved to be highly successful in early (category I) BU cases. Once treated with appropriate antibiotics, there was a very low rate of BU relapse [11]. Clinical trials of oral treatment for the early stages of BU using rifampicin and clarithromycin have been found promising and recently approved for use by WHO [3]. The key to community management of BU is identifying cases in the early stages of the disease and enrolling the afflicted in treatment programs with minimal delay and sustained adherence to treatment.

**Fig 1:**
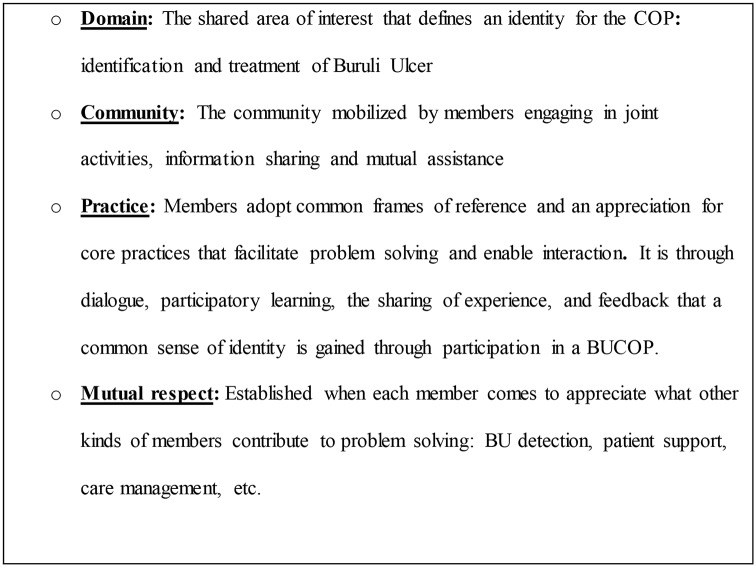
Four essential characteristics of a BU community of practice: Domain, community, practice, mutual respect.

In this paper, we describe a pilot project conducted in Bankim District, Cameroon that proved to be highly successful in establishing a BU community of practice (BUCOP) (Fig 1). A community of practice (COP) is an assemblage of stakeholders committed to a common objective, a common basic understanding of a focal problem, and mutual respect for what each stakeholder contributes to a process of problem solving [12,13]. In the case of BU, this entails health staff, community health workers (CHWs), and traditional healers sharing a common understanding of the signs of BU, collaboration in encouraging the afflicted to seek and continue BU treatment, open lines of communication between stakeholders, and mutual respect for what each contributes to a process of healing that includes, but extends beyond the management of BU as a disease.

## Methods

### Setting

**Fig 2:**
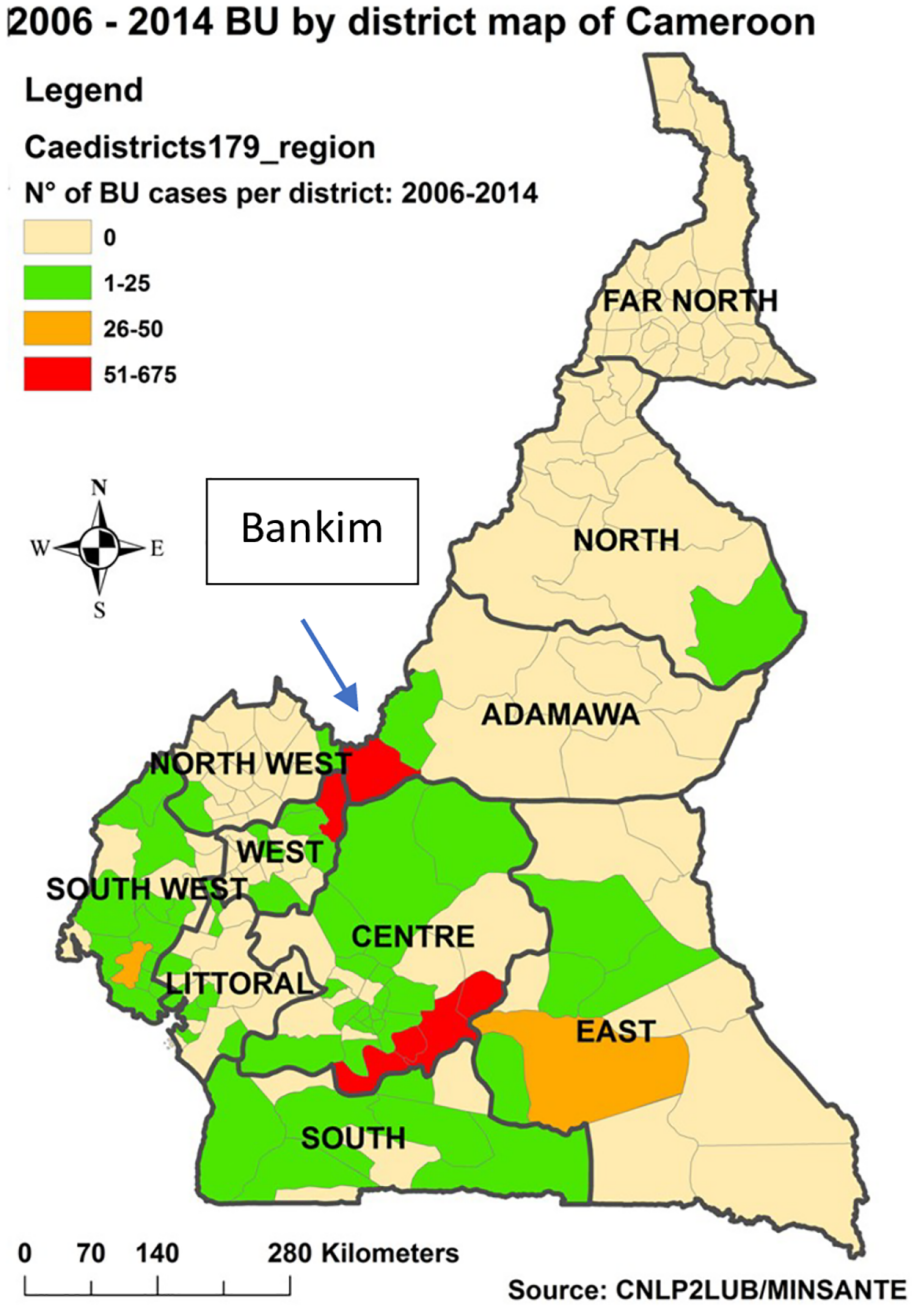
Cases of Buruli ulcer by district of Cameroon 2006–2014.

BU has been identified in 64 of Cameroon’s 179 districts. Bankim District, the focus of this paper, is located in the northwest Adamawa region of Cameroon bordering Nigeria. As noted in Fig 2, the district has one of the three highest prevalence rates for BU in the country [14].

The central treatment and referral hospital for BU in Cameroon is the Ayos District Hospital located in the southern part of the country. Until recently, BU patients from all over Cameroon had to travel considerable distances to be treated at Ayos, a journey many were reluctant to make. Bankim is located over 475 KM away from Ayos and over 10 hours by local transport. Over the last decade, Cameroon’s National BU Control Program has trained clinic staff to provide treatment for BU in its early stages in many regions of the country and established five diagnostic and advanced treatment centers. Another two BU treatment centers are soon to be functional [14].

Bankim is located in a remote region of Cameroon with environmental factors favoring the presence of the MU microorganism responsible for BU. The district is situated in the Mape River Valley, where a dam was built to generate hydro-electric power more than 25 years ago. The Mape Dam splits the area into isolated islands and scattered villages. In the last two decades, increased irrigation has enabled rice cultivation. Rice farming has been identified as a possible risk factor for BU transmission [15,16]. Inhabitants of the region also engage in the growing of maize, cassava, and peanuts as well as various forms of hunting and fishing. Much agriculture is done on plots of land some distance from villages during the months of January to May, and many inhabitants seek employment in Nigeria from November to April. Population movement is both fluid and seasonal.

Health services in Bankim district include a district hospital and 5 satellite clinics. The hospital, at the onset of the project, was staffed by one doctor, 2 nurses, 4 nurse assistants, and one lab technician. The 5 satellite clinics were staffed by one nurse and 1–3 assistants. Two of the 5 satellite clinics had a lab technician conducting basic laboratory analysis. Each large village in Bankim has one community health worker/ volunteer (CHW) who assists hospital staff with outreach activities when requested to do so. There are 86 primary schools in the district and 10 secondary schools. School attendance waxes and wanes depending on season and agricultural activities.

Bankim Health District is a challenging place to initiate a community outreach program for BU due to both its rugged terrain and the wide variety of ethnic groups inhabiting and moving in and out of the region. These groups speak a variety of languages and dialects in addition to French and Pidgin English. The region is inhabited by Tikars, Yambas, Mambilas, Kwanjas, Fulanis as well as ethnic groups hailing from the neighboring Western, North West, Central, and Adamawa regions of Cameroon, and Nigeria. This required BU outreach activities to be carried out in multiple languages and for the research team to seek the approval and support of Christian and Muslim clerics, influential traditional healers, and local chiefs. There are seven paramount chiefs responsible for the welfare of Bankim district. The project staff had to gain the permission from each paramount chief before they could initiate outreach activates in their domain. A proof of concept study was first initiated in the jurisdiction of the paramount chief of Bankim town, a forward thinking, but cautious leader. Once the project was deemed feasible, other chiefs agreed to sanction community based activities conducted during the pilot phase of the project.

### Formative research and phases of research

This community-based intervention employed a seven-stage formative research process [17] summarized in (Table 1) and adapted for BU. The formative research process covers all aspects of an intervention from the collection of baseline data and problem recognition to the generation and weighing of possible interventions from the vantage point of different stakeholders to project implementation, monitoring and evaluation.

**Table 1:** Stages of formative research adapted for a community- Based Buruli ulcer intervention.

Stages of Formative Research 1–7			
**Stage One and Two** **Research “Why” and “What” Questions**	**“Why” questions** Why don’t villagers come for free Bu treatment? Why do they come late? Why don’t they complete therapy or drop out? Why do they consult traditional healers for chronic wounds?	**“What” questions** What predisposing factors influence health care seeking (HCS) for BU? What local illness perceptions and practices influence HCS for BU? What enabling factors influence HCS for BU? What seasonal and livelihood related factors influence HCS for BU?	**“What” questions about health services** What are local concerns related to health service utilization for BU What are local perceptions, rumors, concerns about BU treatment What are local perceptions of government health services: trust, fears What is staff and health volunteer motivation for engaging in BU outreach
**Stage three** **Research “How to” questions related to health service research problems**	**How can we:** Identify BU in early stages when BU is easiest to treat? Make BU programs more accessible and acceptable?	**How can we:** Better enable patents to follow the course of BU therapy as recommended? How can we be more innovative and effective in delivering BU services?	**How can we:** Motivate community health workers to be more proactive in BU outreach? Involve traditional healers in BU programs as partners and not see them as obstacles?
**Stage four** **Identify and assess BU intervention options**	**Generate options** Intervention options identified with key informants	**Conduct SWOT analysis** Assess the strengths and weaknesses, opportunities and external threats of each intervention option	**Assess differential responses by stakeholders** Stakeholders having different needs and vested interests (patients, household members, CHW, health care providers, hospital administers, and so on)
**Stage five** **Research “How to” best implement most promising interventions**	**Asses option feasibility** Consider the pragmatics of implementation	**Pay attention to details** Who, when, where, and how much How best to introduce, organize, support shifts in roles	**Exploration of supportive collaborations** Identify likely challenges and bottlenecks to task sharing and task shifting
**Stage six** **Monitor interventions**	**Monitor options implemented in real time** To facilitate mid-course correction and refinement	**Create opportunities for feedback** Meeting of stakeholders How to foster communication and dialogue	**Facilitate Problem solving process** Identify best means of conflict resolution when such is necessary
**Stage 7** **Evaluate short term effectiveness and long term impact of interventions**	**Outcome evaluation** Evaluate intended outcomes over time Community awareness of BU Referral patterns Data on suspected and confirmed cases of BU identified by community stakeholders—by category of disease severity Data on treatment adherence and so on	**Process evaluation** Evaluate new lines of communication and cooperation Evaluate task shifting and task sharing; and shifts in social roles and relations between BUCOP members Do different stakeholders see such shifts as positive or negative? Do shifts in social relations better enable outreach activities and enhance patient? How has conflict resolution processes in place worked?	**Impact evaluation** Evaluate whether community stakeholder involvement in BUCOP has been sustained over time and can be leveraged Does collaboration extend beyond BU? Has social status of CHW and reputation of hospital improved? Are healers seen as partners in health programs; how has their status in community and health service changed? Have positive relationships been sustained; once pilot project has ended, what minimum resources will be necessary to keep relationships strong Has the BUCOP served to add a community component to health service strengthening efforts? Can new relationships in BUCOP be used as a base for preparedness and emergency response to emerging diseases like Ebola?

The three phases of the project are summarized in Table 2. Phase one focused on baseline data collection, problem identification, and the generation and assessment of intervention options. Phase two had two parts. Part one entailed a proof of concept study to test the feasibility of promising interventions on a small scale. Part two applied lessons learned in the proof of concept study to a large pilot study. In phase three, outcome, process and impact evaluations of the pilot intervention were carried out.

**Table 2:** Three phases of COP research following seven stage model of formative research.

Research phase	Formative research stage	Year	Core activities	Role of social scientist
**Phase one** Baseline data collected prior to intervention Identify problems Generate and weigh intervention options, Work out details of intervention strategies	Formative research stages 1–4	**2010–2011**	Three social scientists conduct focused ethnographies related to BU Former BU patients and community members Health system and staff Community health workers Traditional Healers	Ethnography Health service Research Action research to identify potential intervention strategies
**Phase two (two parts)** **Proof of concept study** (POC) Intervention implementation and refinement **Pilot study (PS**) Intervention implementation, monitoring, problem solving	Formative research Stage 5–6	* POC 2011–2012 * PS 2013–2015	**POC:** Implementation of intervention in early stages Monitor and make suggestions for mid- course correction **PS** **Year one:** social scientists facilitate implementation **Year two:** Hospital and clinic staff take over implementation	**POC:** Implementation research; social scientists as facilitators **PS** **Year one**: social scientists facilitate **Year two**: **Social scientists monitor only**
**Phase three** **Evaluation**	Formative research stage 7	2016	Outcome evaluation Process evaluation: shifts in social relations, partnerships, task shifting and task sharing, role identity, new norms and practices Impact evaluation: impact on stakeholder relations and health care seeking beyond BU	Ethnography: Interviews, case studies, observations of stakeholder meetings, focus groups Data triangulation from mixed methods Evaluation (Qualitative and quantitative data)

In the first phase of the project, a team of three Cameroonian anthropology graduate students and their research advisers conducted interviews with current (N = 69) and former BU patients (N = 22) as well as health staff (N = 15), traditional healers (13), and CHWs (19). These interviews probed predisposing, enabling, and health service related factors influencing health care seeking for chronic ulcers, and reasons for treatment delay and drop out among BU patients offered free treatment. The team identified barriers to treatment adherence related to: 1) cultural perceptions of why wounds do not heal in a timely manner; 2) fear of hospital treatment and trust in hospital staff, and 3) pragmatic issues such as poor transportation, housing, and the availability of food for patients and caretakers when treatment requires hospitalization. The three anthropologists were then embedded in separate communities to investigate the current role of community health workers and healers in chronic wound management, existing BU detection activities, patterns of treatment referral, and household wound care decision making in different seasons. Health staff interaction with BU patients, CHWs, and healers were also observed in the community, at the district hospital, and in local clinics. Research methods employed included participant observation, key informant interviews, prospective and retrospective case studies of BU patients, semi-structured interviews incorporating “what if” scenario, and observations of social interactions between health staff and community members.

At the end of four months of intensive ethnographic research, the anthropological team presented their findings at a workshop attended by the doctor in charge of Bankim district hospital, the heads of the Cameroon National BU Program, and representatives of the NGO Fairmed providing support for neglected tropical diseases (NTD) programs in the region. In keeping with stages 2–4 of formative research, the team also presented data on what different stakeholders saw as possible means of more proactively involving community members in BU detection and the kinds of support that might reduce treatment delay and drop out.

Two major concerns were raised at the workshop. The first concern entailed the need to pay respect to local culture while at the same time addressing cultural beliefs and practices that posed barriers to BU detection and treatment. This required gaining the trust of local leaders and traditional healers and enlisting their support in a new BU outreach initiative. The key question posed was: Would it be possible to involve traditional healers in community-based BU outreach such that they become part of the solution, rather than a major part of the problem of BU treatment refusal, delay, and drop out? The second concern raised was pragmatic. What could be done to reduce the difficulties faced by impoverished patients faced with having to travel long distances to clinics for daily outpatient treatment or required to remain at Bankim hospital as an inpatient for months? The question posed was: would providing transport to the clinic, and food and lodging when necessary, increase the local population’s willingness to seek BU treatment early and adhere to treatment guidelines?

Phase two of research entailed a small- scale proof of concept (POC) study testing the feasibility of a package of proposed interventions to enhance BU outreach and establish a BU community of practice. The twin objectives of the intervention were to raise consciousness about BU using mass outreach events, and to use these events as an opportunity to establish collaborative relationships between clinic staff, chiefs, CHW, traditional healers, and recent BU patients who had a positive treatment experience. A second part of the POC was testing different types of patient support.

The POC study produced positive outcomes (reviewed in the Results section) warranting a larger scale pilot study in Bankim district. In the second part of phase two, a three year pilot study was launched after four modifications were made. The first modification entailed the use of a new WHO promotional video for BU in outreach programs prior to presentation of the educational PowerPoint. Videos are very popular in rural Cameroon. Although the new video was not designed for educational purposes, the theme of hope portrayed was in line with the BUCOP outreach program. Research revealed that while community members did not comprehend much of the language contained in the video, they were happy to see images of patients who had recovered from BU after treatment. The messages in the video did not duplicate nor clash with the messages presented in the educational power point presentation.

A second modification was using CHWs to engage in simultaneous translation into local languages. Hospital staff would present PowerPoint slides in French while a community health worker would translate the messages into local languages (Fulani, Pidgin English, Kwanja, Mambila, Yamba, and Tikar). Teams of presenters adopted a familiar presentation style commonly used in Pentecostal churches with messages passed back and forth between languages in a free and easy style. Pilot research revealed that although repetitive for speakers of multiple languages, translation was responded to positively by audiences even though it increased the duration of the program.

A third modification involved expansion of the number of traditional healers involved in the BUCOP. The healers participating in the POC were carefully screened as exemplars to model best practices. In the pilot study, three other healer groups located in the district were invited to participate in the BUCOP. Members of these healer groups were trained using the BU PowerPoint presentation as a common reference point for instruction. Healer groups met every month to discuss cases and each group monitored members’ adherence to a BUCOP contract. Traditional healers were also invited to participate in community outreach activities in their locales, often working along with CHWs. As in the POC study, they were paid a small honorarium for their efforts.

A fourth modification entailed an upgrade of the halfway houses and hospital wards. During the POC, concern was expressed about unhygienic conditions in the temporary halfway houses. Two halfway houses with bore wells were constructed in rural areas and visited by health staff traveling by motorcycle. At Bankim hospital, both a BU ward and a well laid out dressing room were constructed. A second surgeon was stationed at the hospital such that most skin grafts could be carried out in Bankim rather than being referred to Ayos hospital.

As noted in Table 2, the role of the team of anthropologists shifted over the course of the pilot project. During the POC and the first year of the pilot study, the anthropologists played an active role assisting in the implementation of the intervention package and monitoring interventions, enabling mid-course correction. Anthropologists acted as change agents and were consulted by clinic staff when problems arose. Gradually, implementation of all intervention activities was turned over to hospital staff, CHWs, and leaders of traditional healer groups. By the second year of the pilot study, anthropologists assumed the role of participant observers, monitoring outreach and referral activities, and documenting cases of successful partnerships as well as the ways in which members of the BUCOP solved problems. Then, in the third year of the pilot, the social scientists left Bankim for 12 months to see if BUCOP activities would be sustained without their presence as cultural brokers.

Phase three of the project took place one year after the social science team left Bankim. The team returned and conducted outcome, process, and impact evaluations to assess the effectiveness of the intervention in terms of BU detection, treatment adherence, and BUCOP stakeholder collaboration without the presence of social scientists as change agents. Attention was focused on whether COP member partnerships and lines of communication were sustained.

During this evaluation phase of the project hospital records were reviewed and 44 interviews and twelve focus groups were carried out with 22 CHWs, eighteen healers, nine health staff, nine former and eight recent patients, and five government administrators responsible for health activities in the district. The three anthropologists who had carried out stage one formative research investigated shifts that had occurred in community stake holder relationships with clinic staff as well as each other, task sharing, and changes in the social status of health staff, CHWs, and traditional healers. Broad impacts of the project were assessed as well. Chief among these was whether collaborative relations established by the BUCOP were being leveraged and extended to other health initiatives, and whether the BUCOP model constituted a viable means of promoting trust between health center staff and community stakeholders.

### Ethical considerations

Ethical clearance and research authorization for the project was secured from the National Ethics Committee of the Cameroon Ministry of Health. The District Medical and Sub-Divisional Officers granted authorization for local entrance into the region and community leaders beginning with the Paramount Chief of the Tikars approved of the project. Informed consent was obtained orally from all adult participants in the project after being assured that their participation was voluntary, that the information they shared was confidential, and that they had the right to decline to be interviewed at any point during the project. Oral consent was necessitated given both low rates of literacy and the need to communicate details about the project in local languages, some of which are only spoken.

## Results and discussion

Research results are presented by phase of research.

### Phase one

Four sets of observations made during phase one formative research may be briefly highlighted: 1) local perceptions of chronic ulcers encompassing BU, 2) health care seeking patterns for chronic ulcers, 3) health staff, CHW and healer interactions; and 4) problems in BU identification and treatment warranting intervention.

### Local perceptions of chronic ulcers

Chronic ulcers that do not heal, a hallmark of BU, are often but not always attributed to the local disease category *Mbouati* (*Atom* in other parts of Cameroon) [18,19,20] a spirit affliction that is also the sign of special power accorded to the afflicted. Informants across all ethnic groups voiced the opinion that both *Mbouati* and BU co-existed in the region, some believing that only healers could determine the difference between the two and transform *Mbouti* into a chronic physical ulcer (*nbong*: a widely used Tika term) amenable to successful treatment. A common perception was that dual illness causality could be responsible for ulcers that do not heal. Such ulcers could either be caused or complicated by a combination of natural and supernatural factors [20]. There was also widespread speculation that in recent years the type of ulcer health staff call BU had increased in the region following the dam project and the introduction of rice cultivation.

### Health care seeking for chronic ulcers

Traditional healers are very popular in Bankim and often turned to as a first source of treatment for skin lesions, especially if they do not heal and are linked to witchcraft or *Mbouati*. Healers commonly use herbs, incantations, talismans, and “vaccination” (cutting, burning wounds, etc.) to treat chronic ulcers. While healers work independently, many are members of healer groups, which answer to the paramount chief of Bankim, recognized to be the chief of healers in the district. Healers have close ties to village chiefs and are called upon to offer blessings and protection to the community.

The hospital was not a place that villagers commonly readily turned to for treatment of BU for three reasons. Community members were afraid of hospital based BU treatment as it was associated with operations and amputation. Second, they had little interest in being referred to Ayos hospital as it was far off and in a place foreign to them. Third, despite BU treatment being free, people feared the indirect costs of treatment and hospitalization. There was also some confusion about what kind of wounds were being treated free. While medicines for BU were supplied free of cost by the Cameroonian NTD program, other chronic ulcers that look like BU are not treated free.

### Health staff: Healer and CHW relations

Healers did not have close working relations with clinic staff and did not refer cases to them. In the five years prior to the research project, not one case of BU had been referred to Bankim Hospital or any satellite clinic by a traditional healer. When healers visited Bankim hospital at the request of patients, they did so secretly. Healers also had little contact with CHWs.

CHWs attended meetings at the district hospital when called to do so, but their role was passive. They were given standard WHO educational materials depicting the signs of BU, but were not involved in actively educating community members about BU. For the most part, CHWs only identified possible BU cases when the disease control officer (DCO) from Bankim hospital personally visited their village by motorbike and directly asked CHWs to be shown villagers with chronic wounds. In the five years prior to the research project, CHWs identified only 48 potential BU cases and all of these cases were in advanced stages. BU cases were detected by chance by health staff during vaccination campaigns, and by the DCO when assisting a foreign research team in identifying cases for a clinical trial testing thermotherapy as a possible means of treatment [21]. CHWs did not see themselves as having a clear role in organizing BU outreach activities and they were not in close communication with clinic staff. Health staff saw BU detection and treatment as the responsibility of the DCO and surgeon who headed Bankim hospital.

### Interventions suggested by formative research

Four types of interventions were called for on the basis of formative research. First, a new outreach education program (described below) was needed to raise community awareness about BU and its treatment, address rumors undermining confidence in clinic based care, and foster hope as a means of diffusing fear about BU treatment. Second, CHWs and traditional healers needed to be mobilized, and given a proactive role in both BU case detection and referral as well as patient follow up and psychosocial support. Third, visiting a clinic for 56 days of treatment was challenging for members of many households due to travel difficulties and indirect costs. It was clear that education alone was not going to solve the problem of treatment delay and drop out. Transport, and when necessary, lodging and the feeding of patients and their caretakers was required. Fourth, more advanced BU patients unwilling to travel to a referral hospital like Ayos needed to be treated at Bankim Hospital. Upgrades in BU care needed to be made at the hospital and outreach programs needed to inform the local population that high quality treatment for BU was now available locally at the Bankim district hospital.

### Phase two, part one: Proof of concept study

An intervention package addressing these four intervention priorities was developed after options were weighed in keeping with stage four formative research. The first component of the package, seen as the cornerstone for building a BUCOP, was the introduction of a culturally sensitive community-based BU outreach program requiring clinic staff and community stakeholders to work closely together. An innovative education program was already in the process of being developed by teams of West African social scientists participating in the Stop Buruli Consortium, including the team from Cameroon. The education program developed and tailored for each consortium country (Benin, Cameroon, Ghana) is the subject of a forthcoming publication. In brief, it took the form of an image-rich PowerPoint presentation on BU delivered by local teams equipped with portable generators, laptop computers, LCD projectors and sound systems. The outreach program adopted a question–answer format enabling new issues to be added as they arose. Outreach meetings were interactive, not passive, and questions were invited from community members in attendance. As such, the educational presentation was the product of an iterative process. The social scientists investigated how best to respond to questions in a way that was at once scientific and understandable to local audiences. Messages and visuals were tested and changed as needed. Table 3 briefly summarizes the major sections of the PowerPoint presentation (available at https://www.fairmed.cm/defis/maladies-tropicales-negligees). Different messages were designed to inform and educate the community about BU, reassure community members about the quality of care available at clinics, offer hope of a cure, or display stakeholder collaboration.

**Table 3:** Format of outreach education.

Nine sections of outreach education program	Key messages conveyed	Issues downplayed or emphasized
Signs and symptoms of BU, how to recognize the disease, and the need to treat it early	Visuals of physical signs of BU in different stages Visual and tactile cues suggesting that a lesion, abscess ulcer or edema may be BU Progression of disease if not treated	Category I and II BU depicted, but not category III as this evoked great fear
High risk environments and modes of transmission	High risk environments where one is more likely to be exposed to Mycobacterium ulcerans Focus was on addressing incorrect ideas about BU transmission and contagion	Less time and attention allotted to risk environments and possible modes of transmission as the science is inconclusive and behavior change related to exposure to water sources difficult given the local reality
What clinic staff do to determine if the affliction is BU or some other disease	Why health staff take swabs, what they look for under the microscope, why medicine for BU is specific and not the same as medications used for other ulcers	Step-by-step explanation of what staff is actually doing along with pictures to offset fears and rumors about what they are doing as a means to increase trust
Effective and ineffective treatments for BU	Why 56 days of pills and injections are needed Why herbal medicine for this disease does not lead to a cure even if a wound is dried	Agricultural analogies used to convey the idea that medication is taken beyond treatment for the visible wound, as a means to get at the roots and seeds of BU as a systemic infection in the body Pictures used to show inappropriate treatment, how drying wound is not curing, and effectiveness of medication after herbal medicine has failed to treat the wound
Traditional healers and rapid referral to clinics Emphasis on rapid referral	Positive messages about exemplar healers who recognize signs of BU and rapidly refer patients to clinic after spiritual protection is offered	No message disrespecting local practices as superstitious Respect for traditional healers’ role in offering spiritual protection for those for whom this is a concern
Quality of care at the clinic	Quality of care offered by staff: pictures of what care in the clinic looks like, approachable staff, hygienic conditions, empathetic caretakers, etc.	To offset fear and evoke confidence
Before and after pictures of BU related wounds successfully treated	Pictures of BU treatment, and the healing process at different stages Depict the healing of ulcers on different parts of the body	Pictures depict children and male and female patients of different ages so members of the audience can personally relate
The presentation ends on a note of hope	Testimonials of patients who have been cured speak of their experiences and to the quality of care they have received at the clinic.	Open microphone: some testimonials are planned and others are spontaneous
Questions from the audience	On any topic related to information presented or any other issue related to BU	Open microphone empowers people to speak Questions are recorded and responses to questions assessed as part of iterative process of ongoing research

Over the course of the POC the education program was developed, and pretested. Community outreach education meetings were held in the evenings in eight communities. CHWs were responsible for organizing meetings and inviting chiefs, local healers, and former patients to attend. Programs were treated as social performances where roles in the BUCOP were enacted. Chiefs and healers were seated in places conveying respect, and they were invited to voice their support for the program using a microphone, itself a symbol of power. Social scientists trained the DCO to deliver the PowerPoint education program during the POC and were on hand to assist in responding to questions from the community.

After the program was complete, hospital staff were then on hand to screen community members for wounds they suspected might be BU. During the POC study, 21 suspected cases of BU were identified by health staff either during or a few days following outreach meetings, (see Table 4 below). Patient’s samples were collected either using wound swabs or fine needle aspiration (for oedematous lesions). Of these, 19 (90.5%) were confirmed to be BU by Ziehl-Neelsen staining and/or polymerase chain reaction tests PCR), of which 10 (53%) were category I–early category II BU cases. In villages too remote to reach with audio and visual equipment, CHWs delivered key messages from the PowerPoint presentation orally, using posters and other visual aids depicting the signs of BU, and holding interactive question–answer sessions. Of cases referred to health staff by CHWs, 40% were deemed unlikely to be BU based on visual inspection. Of the remaining cases referred to health staff, 44 (62%) were confirmed by laboratory test to be BU and treated. Nineteen of these cases were either category I or early category II BU.

**Table 4:** Case referral, confirmation by community stakeholder.

	Community of Practice Stakeholder	Community Health workers**	Traditional Healers***	Former patient	Self/family initiated****	Health staff *****	Total
2011–2012	Suspected	60	49	17	98	21	245
	Confirmed *	37	13	11	45	19	125
2013–2015	Suspected	78	59	16	145	18	316
	Confirmed	23	22	6	81	6	138
Total	Suspected	138	108	33	243	39	561
	Confirmed	60	35	17	126	25	263

* Suspected cases are cases referred to health staff that they deemed to be possible BU based on visual inspection warranting diagnostic testing ** Cases detected during outreach program activities *** Cases detected when healer treats patient **** Cases self-identified within households after exposure to BU outreach program. Note: CHWs and healers are often consulted for a second opinion. Second opinion not presented in this table *****Cases identified by health staff during routine health care activities at clinic or in community (i.e., during immunization programs)

Following POC outreach educational activities, many cases of chronic ulcers and skin lesions were brought to the attention of CHWs or health staff by community members themselves. Ninety-eight of these cases were suspected to be BU by health staff, of which 45.9% were confirmed a by laboratory test and treated as BU. Of these cases, 25 were category I or early category II BU**.** As a point of comparison, between 2009 and 2010 no cases of BU like symptoms had been self-referred to clinic staff by community members. During the POC study, former BU patients were also encouraged to refer suspected cases of BU. They referred 17 cases to health staff, of which 11 (64.7%) were confirmed to be BU and treated.

To gain a sense of just how effective the POC was in detecting cases of BU, it is useful to compare POC results with the results of a house to house NTD survey conducted in Bankim district between late March and mid-April 2010. During the NTD survey researchers visited 9,344 households (48,962 people). The survey only identified 25 suspected cases of BU, of which six were confirmed to be BU by PCR [4].

A second component of the POC study was provision of free transport to clinics for category I and early category II BU patients not requiring hospitalization and living 3–7 KM from clinics. Motorcycle taxis were hired to deliver BU patients to the hospital for the duration of treatment. For those who lived too far to make this feasible, housing was secured for them near clinics in facilities termed halfway houses. Halfway houses were tested in both Bankim town and two rural areas. In Bankim town, patients staying at halfway houses received treatment and hospital staff routinely monitored their wounds. In the rural areas, local clinic staff traveled 8–10 km daily by motorcycle to administer treatment to patients at halfway houses. Patients and caretakers remaining at halfway houses were supplied a food ration. Seventeen patients took up residence in halfway houses during the POC. Anthropologists monitored social interaction between patients of different ethnic groups and found that they bonded around the shared experience of BU and supported one another during treatment.

The third component of the POC was assessing whether traditional healers would participate in BU outreach activities and become proactive members of a BUCOP. In brief, after obtaining support from the paramount chief of Bankim town, a meeting was held with a group of popular traditional healers who were members of a healer association. Ten traditional healers were selected to participate based on their willingness to collaborate with clinic staff in addressing both biomedical and traditional medical aspects of BU treatment. Clinic staff explained to healers that in order for their treatment to be effective, patients needed to be referred to them quickly and wounds needed to be left undisturbed and not treated with traditional medicines. Clinic staff acknowledged that they had no expertise in the mystical aspects of treatment such as removing spiritual affliction, protecting the patient when vulnerable to forces of malevolence, nullifying obstructions to the healing process, or dealing with patient fears of malevolent spirits during treatment. Healers were asked to attend to the spiritual and psychosocial aspects of treatment, while clinic staff used medications and bandaging to take care of the physical manifestations of the disease.

A contract was proposed and signed by members of this group of traditional healers. Two key components of the contract were that traditional healers promised to refer all patients with possible signs of BU to Bankim clinic staff within ten days of seeing them and not treat the skin of these patients. Clinic staff gave healers free access to Bankim hospital, satellite clinics, and halfway houses to offer patients psychosocial support and spiritual protection. Healers were also offered a small amount of money to pay for accompanying patients to a clinic for screening.

The social science team monitored traditional healers’ adherence to the contract. All ten traditional healers adhered to the contract, and six of the ten healers **referred** 49 suspected BU patients to Bankim hospital. Of the 49 suspected cases referred, 13 (26.5%) were confirmed to be BU by laboratory test. In the case of traditional healers, all cases referred were tested, even when staff thought the case was unlikely to be BU. This policy was followed after a healer challenged a case clinic staff dismissed as not being BU based on visual inspection. The healer was proven correct in her detection of BU.

The treatment adherence rate during the POC for BU patients detected was 94% compared to a rate of 54% in Bankim Hospital in 2009–2010. Increased adherence was due to both enhanced patient support by health staff, CHWs, and traditional healers and the provision of resources better enabling patients to remain in treatment. Notably, all BU patients referred by healers and visited by them in the hospital completed treatment.

### Phase two, part two: Pilot study

During the pilot project an additional 89 CHWs and 55 healers were trained in BU detection. Forty-four well attended community outreach programs were conducted reaching approximately 15,500 people. In Tables 4 and 5 we present data on BU case referral and confirmation by stakeholders during both the POC and pilot interventions. The number of cases referred designates cases referred to health staff and deemed to be possible cases of BU by visual inspection. Confirmed cases were by Ziehl-Neelsen staining and/or polymerase chain reaction tests (PCRs). During the POC approximately 40% of total cases brought to the attention of health staff were dismissed as some other kind of skin lesion, abscess, or ulcer. During the pilot study this percentage dropped to 30% of all cases reported. Table 6 presents data on the category of confirmed BU cases referred by different stakeholders in the BUCOP. Table 7 presents data on adherence to treatment by confirmed cases of BU that initiated therapy. This data is present by the type of stakeholder who referred the case.

**Table 5:** Percentages of cases referred and confirmed by stakeholder.

	Community of Practice Stakeholder	Community Health workers	Traditional Healers	Former patient	Self/family initiated	Health staff	Total
**2011–2012**	**Suspected (N = 245)**	24%	20%	7%	40%	9%	100%
	**Confirmed (N = 125)**	30%	10%	9%	36%	15%	100%
**2013–2015**	**Suspected (N = 316)**	25%	19%	5%	46%	6%	100%
	**Confirmed (N = 138)**	17%	16%	4%	59%	4%	100%
**Total**	**Suspected (N = 561)**	25%	19%	6%	43%	7%	100%
	**Confirmed (N = 263)**	23%	13%	6%	48%	10%	100%

**Table 6:** Category of confirmed BU cases by stakeholder referral *.

	Community of Practice Stakeholder	Community Health workers	Traditional Healers	Former patient	Self/family initiated	Health staff	Category total
**2011–2012**	**Category I**	7 (28%)	3 (12%)	1 (4%)	10 (40%)	4 (16%)	25 (100%)
	**Category II**	12 (28%	7 (16%)	3 (7%)	15 (35%	6 (14%)	43 (100%)
	**Category III**	18 (32%)	3 (5%)	7 (12%)	20 (35%)	9 (16%)	57 (100%)
**2013–2015**	**Category I**	6 (19%)	7 23%)	1 (3%)	16 (52%)	1 (3%)	31 (100%)
	**Category II**	9 (16%)	7 (13%)	2(4%)	35 (64%)	2 (4%)	55 (100%)
	**Category III**	8 (16%)	8 (16%)	3 (6%)	30 (39%)	3 (6%)	51 (100%)
**Total**	**Category I**	13 (23%)	10 (18%)	2 (4%)	26 (46%)	3 (5%)	54 (100%)
	**Category II**	21 (21%)	14 (14%)	5 (5%)	50 (52%)	8 (8%)	98 (100%)
	**Category III**	26 (24%)	11 (10%)	10 (9%)	50 (46%)	12 (11%)	109 (100%)

* Cases confirmed by Ziehl-Neelsen and/or PCR

**Table 7:** Adherence rate of confirmed cases undergoing treatment by stakeholder referring.

Adherence to Treatment	Community of Practice Stakeholder	Community Health workers	Traditional Healers	Former patient	Self/family initiated	Health staff referrals	Total
2011–2015	# of cases treated	60	35	17	126	25	263
	% who completed therapy	93%	86%	94%	97%	92%	94%

Several findings stand out as notable. First, prior to the project no cases of BU were referred to health staff by community stakeholders with the exception of cases identified when a disease control officer occasionally visited a community searching for NTD cases. During the project, 90% of all confirmed cases of BU were identified by community stakeholders. Second, as a result of the project not only was there a significant rise in the number of BU cases referred by community stakeholders, but a significant number of category I BU cases were detected and treated. Five hundred and twenty-two suspected cases of BU that health staff thought warranted laboratory confirmation were referred by community members. Out of these 522 cases, 266 cases (51%) were confirmed to be BU. Twenty-one percent of these cases were category I. Third, more suspected BU cases were referred by family members following outreach educational programs than any other BUCOP stakeholder. Their referrals accounted for 40% of all referrals of suspected cases of BU during the POC, and 46% of all referrals during the pilot study. In many instances, family members checked with CHWs and traditional healers participating in the BUCOP to ask their opinion about the lesion or to request that they accompany them to a clinic. In the same period, 23% of suspected cases were detected and referred by CHWs, of which 17% were confirmed of which 19%% were category I. Health staff only detected 39 cases of BU during their routine activities, of which only five cases (13%) were category I.

A fourth finding was that traditional healers actively participated in both the POC and pilot project and kept to the terms of the contract they signed. Traditional healers referred 19% of all suspected cases of BU during the pilot project, 15% of all confirmed cases, and 18% of confirmed category I cases. Fifth, the confirmation rate of suspected cases sent for testing was impressive. While 30% to 40% of lesions brought to health staff for inspection were dismissed as other skin aliments, a high percentage of the remaining 60–70% of cases were found to be BU. Of cases tested, 43% of those referred by CHWs, 32% by traditional healers, 52% by former patients, and 52% by family members were found to be positive. The rate of confirmation for traditional healers was lower because unlike the other stakeholders every case referred was sent for testing. If 60%–70% of their cases had been sent for testing after staff screening, their confirmation rate would have been similar to other BUCOP stakeholders. Sixth, treatment adherence rates for confirmed cases referred by all stakeholders were > 90%. One can compare this to a 54% BU treatment adherence rate two years prior to the project.

### Process and impact evaluation

The goal of the pilot project was not just to mobilize individual stakeholders to become more actively involved in BU detection and referral, but to create an interactive and supportive BUCOP. Phase three evaluation research revealed that a functioning BUCOP had indeed emerged in Bankim. Lines of communication between CHWs, traditional healers, and health staff were well established and collaboration in BU outreach activities were ongoing. The status of CHWs increased markedly as a result of CHWs becoming actively involved in arranging outreach education and screening programs for their communities. A closer working relationship with clinic staff empowered them to play a more proactive role in both referring and following up suspected BU cases. The status of former BU patients also changed. The community appreciated their testimonials during large outreach meetings. Former patients came to be seen less as victims and more as survivors having BU treatment experience they were willing to share.

Clinic staff noted being surprised by the large number of BU cases referred by community stakeholders as a result of the project. They came to value the BUCOP, stating that it both enabled them to have a much closer working relationship with CHWs and traditional healers, and it enhanced the reputation of the hospital. One testament to growing trust in the hospital was consultation by members of the Fulani ethnic group. Members of this group had previously been reluctant to seek treatment for BU. Outreach education delivered in pidgin by a trained Fulani community health worker (also a traditional healer) and a few treatment success stories have paved the way for greater contact with this community. Additionally, the director of Bankim hospital has been praised by Cameroonian health officials for forging close working relationships with community stakeholders. Better working relations between health staff and CHW, and increased acknowledgment of each stakeholder’s contribution to BU program success served as important non–financial incentives enhancing stakeholder motivation [22].

A major question posed during phase one of the project was whether traditional healers would be willing to actively participate in a BUCOP. The impact evaluation found that healers are presently seen by clinic staff and health officials as having an important role in community-based BU management. Not only have traditional healers referred cases and honored their treatment contract, but they have routinely assisted CHWs during outreach activities, offered psychosocial support to patients who are hospitalized, and encouraged patients who dropped out of therapy to return to treatment. Health staff are now invited to traditional healer group meetings to discuss cases, and healers are invited to hospital meetings when outreach activities are being planned. Health officials were initially reluctant to give traditional healers identification badges designating them as members of the BUCOP. By the second year of the pilot project, officials felt that healers had proven their commitment to the BUCOP. Traditional healers were offered badges along with referral cards and health officials left it up to traditional healer groups to both monitor member adherence to the contract and to sponsor new healers who wished to join the BUCOP after receiving training.

A key issue investigated was what healers gained and lost from participation in the BUCOP. Some members of the NTD community initially expressed the opinion that traditional healer participation might be primarily motivated by funds received when referring patients. This opinion proved to be a false. Research revealed that healers lost far more financially then they gained by referring patients for treatment. At the time of the impact evaluation, funds for healers to accompany patients to the clinic were exhausted. Yet healers continued to refer as well as visit patients at the clinic without charging any fee in cash or in kind. During healer groups, cases were discussed and when one healer did not have the funds to take a patient to the clinic or visit them, another member of the group often offered to do so.

So what did healers get out of the becoming members of the BUCOP? Research revealed that the social and symbolic capital healers gained outweighed any financial loss they incurred for referring patients. Healers who visited Bankim hospital were warmly received by health staff who saw them as an asset in reassuring patients about their treatment. Healers took pride in offering spiritual protection to patients so that BU medications could act effectively, and for offering patients psychosocial and spiritual support while under treatment. When BU patients were cured, healers shared credit with health staff for treatment success. While healers received no payment for their BU related activities, they did receive gifts when patients were cured. Moreover, other patients residing at the hospital requested their assistance. In short, their reputation increased and was not diminished by collaboration with clinic staff.

An additional impact of the BU project was its contribution to the integration and control of other neglected tropical skin diseases. Although not intended to do so, BU outreach programs attracted a large number of cases of yaws, a disease not seen at clinics in the district for over a decade and assumed to be eradicated in this region of the Cameroon. Cases of yaws identified during BU outreach programs alerted health staff of the presence of the disease and they then conducted follow up school based screenings in these communities. Eight hundred and fifteen cases of confirmed cases of yaws were successfully treated [23].

### Limitations and challenges

We do not claim that the relationships established in the Bankim BUCOP can be formed in all contexts. Context must be taken into consideration. For example, traditional healers in Bankim are organized into groups which exercise some modicum of authority over their members. Such is not the case in all African contexts. Mobilizing individual healers would prove more challenging than mobilizing groups. A challenge that may be briefly mentioned **is** that when the reputation of a health facility rises so do patient load and resource need. In Bankim, the increased reputation of the hospital has drawn patients from outside the district and even neighboring Nigeria. Resources to maintain quality of care will need to be sustained or the reputation of the hospital will decline.

### Conclusion

In a recent review of the impact of two decades of health social science research on NTDs, Bardosh [24] notes that while this research has generated important insights into health care seeking and community response to disease centered programs, it has not been effectively used in program development and implementation. This pioneering study illustrates how a multistage formative research process can contribute to the creation of a BUCOP. We would argue that the COP model of clinic staff–community stakeholder collaboration presented here has great potential for other community-based disease outreach programs in Africa and beyond. It’s inclusion of community stakeholders like healers, volunteers, and former patients extends the COP model recently advocated for establishing productive relationships between local experts, NGOs, ministries of health and government health staff, and foreign advisers [25, 26]. In Bankim, now that collaborative relationships have been established, they are being leveraged for other public health endeavors such as vaccination programs. Establishing a COP also has great potential for emerging disease preparedness. Should a disease like Ebola strike Bankim, collaborative relationships between clinic staff, CHWs, chiefs, and healers will enable a rapid coordinated response. If the 2015 Ebola outbreak in West Africa taught us anything, it is recognition that close ties to community stakeholders constitute an essential part of health system strengthening [27,28,29,30, 31,32]. Establishing trust and lines of communication with community leaders enables swift action and increased opportunities for local problem solving.

## Supporting information

S1 Checklist (DOC)
